# Data-Based Prediction and Stochastic Analysis of Entrained Flow Coal Gasification under Uncertainty

**DOI:** 10.3390/s19071626

**Published:** 2019-04-05

**Authors:** Iftikhar Ahmad, Ahsan Ayub, Nisar Mohammad, Manabu Kano

**Affiliations:** 1 Department of Chemical and Materials Engineering, National University of Sciences and Technology, Islamabad 44000, Pakistan; 2 US Pakistan Center for Advanced Studies in Energy, National University of Sciences and Technology, Islamabad 44000, Pakistan; ahsan_che06@scme.nust.edu.pk; 3 Department of Mining Engineering, University of Engineering and Technology, Peshawar 25000, Pakistan; nisar.mohammad@nwfpuet.edu.pk; 4 Department of Systems Science, Kyoto University, Kyoto 606-8501, Japan; manabu@human.sys.i.kyoto-u.ac.jp

**Keywords:** entrained flow coal gasification, Sobol test, fourier amplitude sensitivity test, uncertainty analysis, sensitivity analysis, polynomial chaos expansion

## Abstract

Entrained flow gasification is a commonly used method for conversion of coal into syngas. A stable and efficient operation of entrained flow coal gasification is always desired to reduce consumption of raw materials and utilities, and achieve higher productivity. However, uncertainty in the process hinders the stability and efficiency. In this work, a quantitative analysis of the effect of uncertainty on the conversion efficiency of the entrained flow gasification is performed. A data-driven, i.e., ensemble, model of the process was developed to predict conversion efficiency of the process. Then sensitivity analysis methods, i.e., Sobol and Fourier amplitude sensitivity test, were used to analyze the effect of each individual process variables on conversion efficiency. For analyzing the collective impact of uncertainty in process variables on conversion efficiency, a non-intrusive polynomial chaos expansion (PCE) method was used. The PCE predicts probability distribution of the conversion efficiency. Reliability of the process was determined on the basis of percentage of the probability distribution falling within control limits. Measured data is used to derive the control limits for off-line reliability analysis. For on-line reliability analysis of the process, measured data is not available so a just-in-time method, i.e., k–d tree, was used. The k–d tree searches the nearest neighbor sample from a database of historical data to determine the control limits.

## 1. Introduction

Coal is one of the major energy sources being used for several centuries. According to British Petroleum (BP) statistical review of world energy, an estimated amount of 1,139,331 million tonnes of reserves exists in different parts of the world [1]. In spite of the concerns raised by environmental agencies and lower usability of coal as a fuel, the shortage of other energy resources makes the use of coal still valid. Entrained flow gasification is a commonly used method for conversion of coal into a green form of fuel, syngas. Extensive work on coal gasification has been reported in literature [2,3,4,5,6,7,8,9,10,11,12,13,14]. A stable and efficient operation of entrained flow coal gasification is a highly desired objective of the researchers to reduce its environmental impact and achieve higher conversion efficiency.

For realizing efficient process design, process modeling of the entrained flow gasification process has been the focus of research [2,3,4,5,8,9,10,11,12,13,14]. Ni et al., in 1995, developed a multivariable model for performance estimation of the entrained flow coal gasifier [8]. Liu et al., in 2000, investigated the effect of char structure and kinetics on the behavior of coal char in a pressurized gasifier [10]. Vicente et al., in 2003, developed a model to analyze turbulent dispersion and coal particle drag in a coal gasification process [11]. Chen et al., in 2012, investigated the effect of inlet flow pattern and coal to steam ratio on the conversion efficiency of the process [14]. Ilamathi et al., in 2013, used artificial neural networks (ANN) to predict unburnt carbon in a gasifier. In addition, a genetic algorithm (GA) was employed to optimize process conditions for realizing reduction in unconverted carbon in bottom ash [2]. Halama et al., in 2016, studied the behavior of two phase mixture, i.e., solid particles and gas phase, in char gasification by using computational fluid dynamics (CFD) techniques [3]. Mitianiec et al. (2017) used CFD simulation for an axial fluidized boiler to determine the thermal loads and toxic emissions during combustion of coal [4]. Wu et al., in 2017, developed a hybrid framework comprised of kinetic model and energy utilization diagram for prediction of reaction progress and exergy destruction in coal gasification [5].

Process uncertainty is a challenge in realizing stable and efficient operation of the coal gasification process. Some work is reported in the literature on analyzing uncertainty in coal gasification process models [9,12,13]. Chen et al., in 1999, used the multi-solids progress variables (MSPV) method to analyze uncertainty in product gas properties; the effect of various feed parameters, like coal type, gas flow rates, and the temperature, was studied [9]. Watanabe et al., in 2006, studied the fluctuations in the coal gasifier’s performance by varying some input parameters, e.g., gasifier pressure, air ratio, and coal type [12]. Similarly, Haung et al., in 2007, performed the sensitivity analysis of coal type, bed temperature, static bed height, and various feed flow rates [13].

In the present work, a novel framework comprising of data-based prediction, sensitivity analysis, uncertainty analysis, and reliability analysis is proposed. A data-driven model based on ensemble technique was developed to predict conversion efficiency of the entrained flow gasification process. For the development of the data-driven model, data was generated through the interfacing of MATLAB^®^–Excel^®^–Aspen^®^. In order to analyze the effect of each individual process variable on the conversion efficiency of the process, Sobol and Fourier amplitude sensitivity tests were used. The two methods, i.e., Sobol and Fourier amplitude sensitivity tests, were used to cross-check their index-based ranking of input variables. Non-intrusive polynomial chaos expansion (PCE) was used to analyze the collective effect of uncertainties on the conversion efficiency. PCE is a stochastic approach that resulted in a predictive distribution of conversion efficiency of the entrained flow coal gasification. The reliability of the process was determined on the basis of the probability distribution falling within control limits. Measured data is used to derive control limits for the reliability analysis. For on-line reliability analysis of the process, measured data is not available so a just-in-time method, i.e., k–d tree, was used. The k–d tree searches nearest neighbor sample from a database of historical data to determine the control limits.

Section 2 presents process description of entrained flow coal gasification followed by modeling methods described in Section 3. The proposed modeling framework is shown in Section 4, and then the results and discussion are provided in Section 5. Section 6 concludes the work.

## 2. Process Description

**Figure 1:**
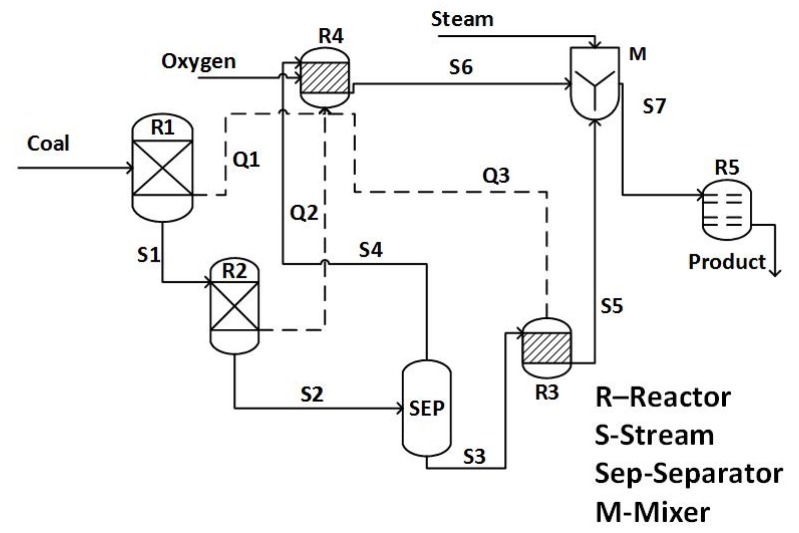
Process flow diagram of entrained-flow coal gasification.

A process flowsheet of an oxy-fired entrained flow coal gasification is shown in Figure 1. This is a schematic representation of an Aspen PLUS^®^ based model derived from the work of Wen et al. [15]. The symbols used in Figure 1 are defined in Table 1. Major process units of the flowsheet were pyrolysis, separator, volatile combustion, and char gasification. The pyrolysis process had two reactors, i.e., R1 and R2, in series. In R1, a pressure drop occurred which was readjusted in R2 to complete the reaction [12]. The outlet of R2 was separated into S3 and S4 by separator, i.e., SEP. The gas stream S4 entered into the combustion reactor R4 along with an oxygen stream. On the other hand, the solid stream S3 went to reactor R3 and decomposed to form C, H_2_, O_2_, N_2_, S, and ash. The mixer M mixed the streams S6 and S5 to form the feed stock for the gasifier R5. The gasifier converts char into carbon dioxide, carbon monoxide, methane, hydrogen, and hydrogen disulfide [12].

Rplug reactor and Ryield reactor, built-in modules of Aspen PLUS^®^, were used for gasification and and pyrolysis processes, respectively. For combustion process and decomposition process, another type of built-in models of Aspen PLUS^®^, Rstoic reactor, was used. A list of reactions occuring in pyrolysis, decomposition, combustion, and gasification processes is shown in Table 2 [15].

In this study, 14 process variables were used as input variables for the development of the modeling framework; see Table 3 and Table 4.

## 3. Fundamentals of Modeling and Analysis Methods

### 3.1. Soft-Sensor Development

**Figure 2:**
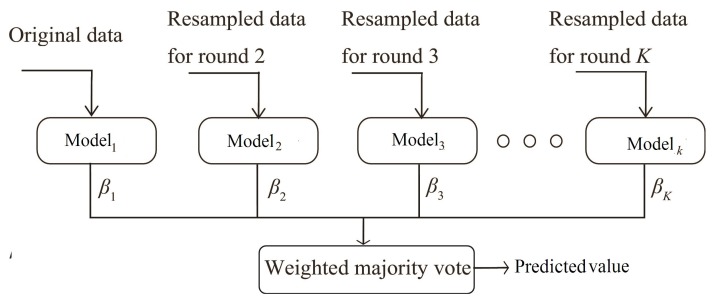
Accumulation of weak models through the boosting technique [17].

A kind of ensemble learning method, i.e., boosting, was used to develop the soft-sensor for prediction of conversion efficiency of the entrained flow coal gasification process [16]. Ensemble learning methods have recently gained the attention of researchers due to their high prediction accuracy and robustness [17]. In the boosting method, several weak models were combined to form a single efficient and robust model as demonstrated in Figure 2 (revised figure from [17]). On each round of developing a model, the data sample difficult in learning was getting more focus through weights allotted by the boosting mechanism. Least squares boosting (LSBoost), a boosting method for regression problems, was adopted in this study [18].

### 3.2. Uncertainty Analysis

Uncertainty analysis is a method used for quantification of collective impact of uncertainty in multiple input variables of a process [19]. Ahmad et al. have done an extensive review on dimensions and analysis methods of uncertainty in process models [20]. Although several methods are available for uncertainty quantification, the current study used a sensitivity analysis method and a sample-based method. The sensitivity analysis method helped in determining the impact of individual variables on the process output while the sample-based method assessed the collective impact.

#### 3.2.1. Sensitivity Analysis

For the sensitivity analysis, Sobol technique and Fourier amplitude sensitivity test (FAST) were used [21,22,23]. The two methods were used to cross-check their index-based ranking of input variables.

Consider a model y=f(x), where *y* is a scalar output and input factors $x_{1},...,x_{p}$ are independent random variables following known probability distribution. In the Sobol method, f(x) is decomposed as follow:

(1) $$f\left(x\right)=f_{0}+\sum_{i=1}^{p}f_{i}\left(x_{i}\right)+\sum_{1\leq i<j\leq p}f_{ij}\left(x_{i},x_{j}\right)+...+f_{1},...,p\left(x_{1},...,x_{p}\right).$$

Furthermore, the variance v(y) is decomposed into

(2) $$v\left(y\right)=\sum_{i=1}^{p}v_{i}+\sum_{1\leq i<j\leq p}v_{ij}+...+v_{1,...,p},$$

where $v_{i}$, $v_{ij},...,v_{1,...,p}$ denote the variance of $f_{i}$, $f_{ij},...,f_{1,...,p}$, respectively.

The first-order Sobol sensitivity indices were derived as follows:

(3) $$S_{i}=\frac{v_{i}}{v(y)}=\frac{v(E\left(y|x_{i}\right))}{v(y)}.$$

In the Fourier expansion, when all terms were mutually orthogonal, the model y=f(x) was expanded in a Fourier series [24].

(4) $$y=f\left(s\right)=\sum_{j=-\infty }^{+\infty }\left\{A_{j}\cos js+B_{j}\sin js\right\},$$

where the $A_{j}$ and $B_{j}$ are

(5) $$A_{j}=\frac{1}{2\pi }\int_{-\pi }^{\pi }f\left(s\right)\cos jsds$$

(6) $$B_{j}=\frac{1}{2\pi }\int_{-\pi }^{\pi }f\left(s\right)\sin jsds.$$

Variance caused by factor *i*, $v_{i}$, in the output was estimated as:

(7) $$\hat{v_{i}}=\sum_{p\in Z^{o}}\wedge p\omega_{i}=2\sum_{j=1}^{+\infty }\wedge p\omega_{i},$$

where $Z^{o}=Z-0$ represents the set of integer numbers except 0. The total variance is given by

(8) $$\hat{v}=\sum_{j\in Z^{o}}\wedge_{j}=2\sum_{j=1}^{+\infty }\wedge_{j},$$

where $\wedge_{j}=A_{j}^{2}+B_{j}^{2}$ is the spectrum of the Fourier series expansion.

#### 3.2.2. Sample-Based Method of Uncertainty Analysis

A sample-based uncertainty analysis method, i.e., polynomial chaos expansion (PCE), was used for uncertainty analysis of the process. In the PCE based method, a random variable x is represented as a function (f()) [25,26]:

(9) x=f(ξ),

where ξ is another random variable. x is decomposed as follows:

(10) $$x=f\left(\xi \right)=\sum_{i=1}^{\infty }\alpha_{i}\psi_{i}\left(\xi \right),$$

where $\alpha_{i}$ is deterministic components while $\psi_{i}$ is stochastic components, and $\psi_{i}$ obeys the following condition:

(11) $$\langle \psi_{j},\psi_{k}\rangle =\int \psi_{j}\left(\xi \right)\psi_{k}\left(\xi \right)p_{\xi }\left(\xi \right)d\xi =0,j\neq k,$$

where the inner product of $\psi_{j}$ and $\psi_{k}$ results in $\langle \psi_{j},\psi_{k}\rangle$, and $p_{\xi }$ is the probability density function (PDF) of ξ.

In this study, a non-intrusive (black box) method was used to determine the mode strengths [27,28].

### 3.3. Reliability Analysis

**Figure 3:**
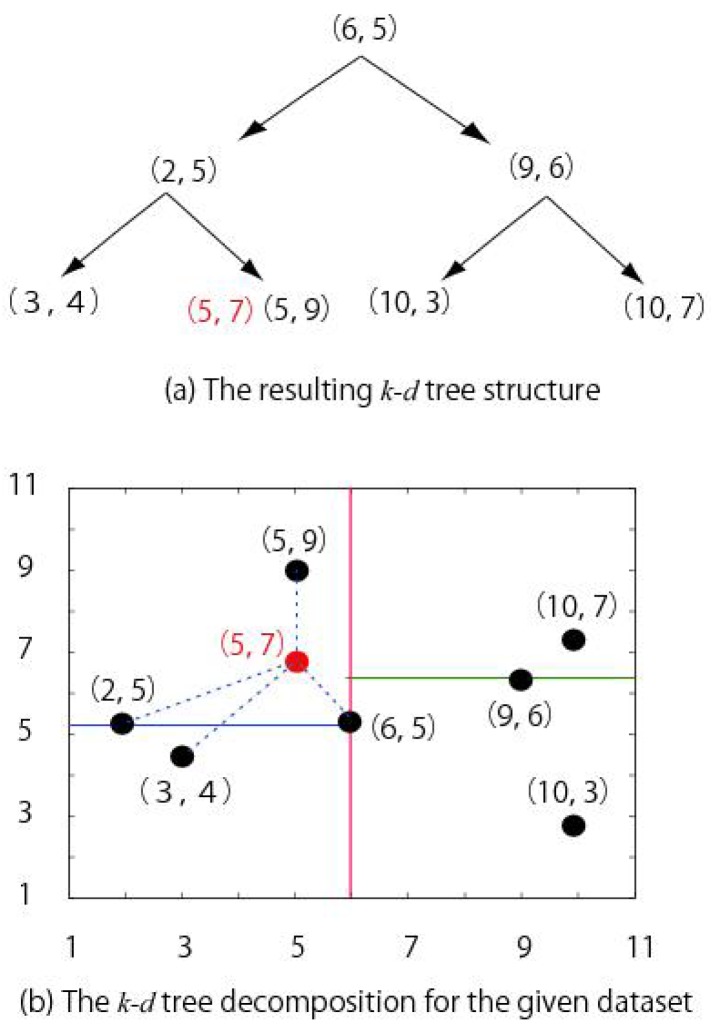
The k–d tree construction [30].

Reliability of a process is very important for stable operation. In this work, a framework of reliability analysis is proposed which monitors the probability distribution of process output, i.e., conversion efficiency, predicted through the PCE based method. Percentage of the probability distribution within control limits is used to asses reliability of process conditions. Off-line reliability analysis was performed by deriving the control limits through measured data of the process. For on-line reliability analysis, a just-in-time method, i.e., k–d tree, was used to derive the control limits. As in on-line operation, measured values of process output were not available so the k–d tree algorithm searches nearest neighbor sample from database of historical measured values [29]. The search for the nearest neighbor sample was carried out on the basis of recursively splitting of data at different dimensions until a child subset with only one sample is left. For a data set comprising of (3,4), (2,5), (5,9), (6,5), (9,6), (10,7), and (10,3), the k–d tree-based search of the nearest neighbor samples for a query (5,7) is shown in Figure 3.

## 4. Modeling and Analysis Framework

The modeling strategy adopted in this work is summarized as follows:

- Data generation: steady state values of the process model were altered through the MATLAB^®^–Excel^®^–Aspen^®^ interfacing to generate data. The data generated through the interfacing was referred to as measured data. We generated 1000 data samples through the interfacing.
- Soft-sensor design: the data was used to develop the boosting based soft-sensor. The number of trees, weak learner, were optimized. The optimized number of trees was 500.
- Sensitivity analysis: to analyze the sensitivity of input variables, a MATLAB^®^-based algorithm of Sobol and FAST techniques was used [22]. The two methods were used to cross-check their index-based ranking of input variables. In both the sensitivity analysis methods, samples were taken for each input variable, listed in Table 3, from a normal distribution of 200 values with a standard deviation of 0.5% of the measured values. Both methods calculated first-order indices of the input variables. The most sensitive input variables were selected and their theoretical basis was analyzed.
- PCE based uncertainty analysis: the ensemble model was incorporated in the PCE framework. Hermite function at level 6 and initial 20 terms was used in the PCE method. The framework was used to generate random variables for each of the input variables. The random variables for inputs of validation samples were fed to the ensemble (boosted) model to get predictive distribution of the process output.
- Reliability analysis: reliability of the process was determined off-line through measured data. For on-line reliability analysis of the process, a just-in-time method, i.e., k–d tree, was used. For the control limits, 1.5% of the nearest neighbor sample values were taken as control limits. A threshold was set for the reliability of the predictive distribution of CO mass flow rate; the process was termed as reliable if 60% of the predictive distribution fell within the control limit otherwise unreliable.

## 5. Results and Discussion

**Figure 4:**
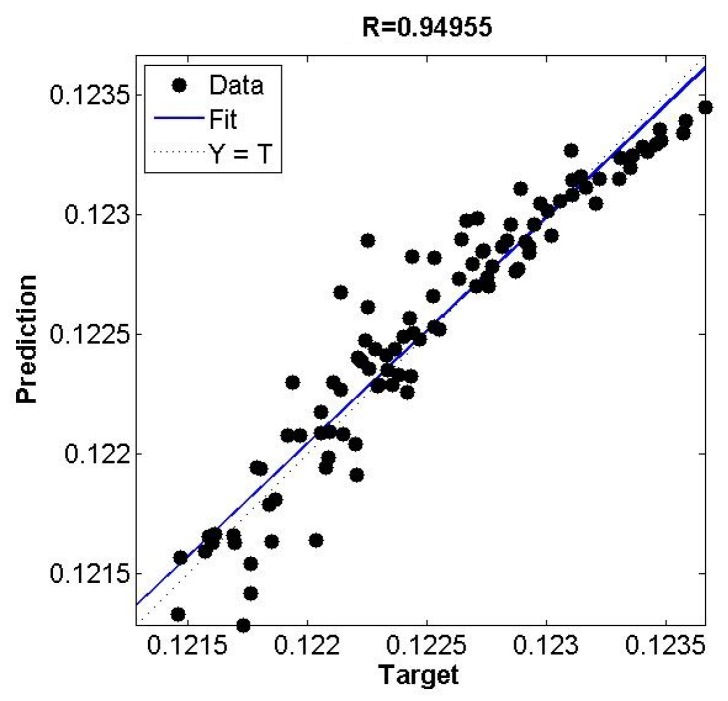
Ensemble model for prediction of mass flow rate (kg/s) of CO.

**Figure 5:**
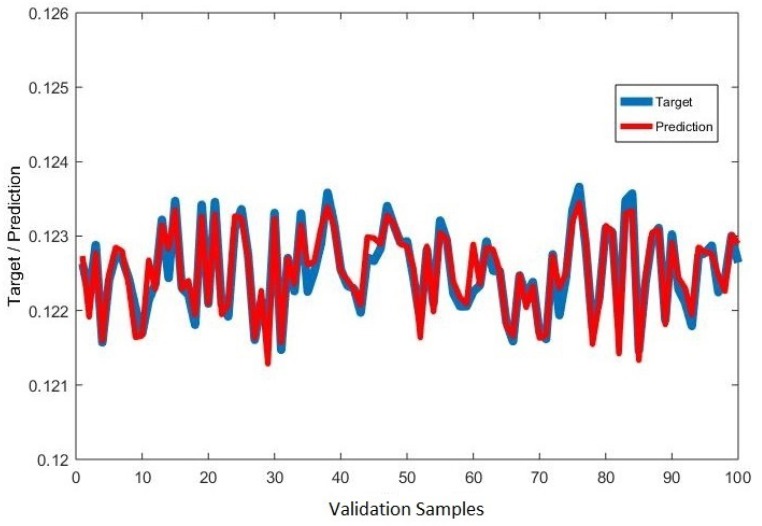
Target and predicted values of mass flow rate (kg/s) of CO.

Considering the fact that CO is one of the desired product components of the coal gasification process, we assumed a mass flow rate of the CO as a representative of the conversion efficiency. A similar assumption has been reported in literature where the mass fraction of CO in product stream is considered as the representative of the carbon element efficiency of the coal gasification process [31]. 90% of data generated from interfacing of MATLAB^®^–Excel^®^–Aspen^®^ was used for model development while 10% was used for validation. Deterministic prediction of mass flow rate of CO through the soft-sensor is shown in Figure 4 and Figure 5. The correlation coefficient between the target (validation samples) and the predicted values was 0.95.

**Figure 6:**
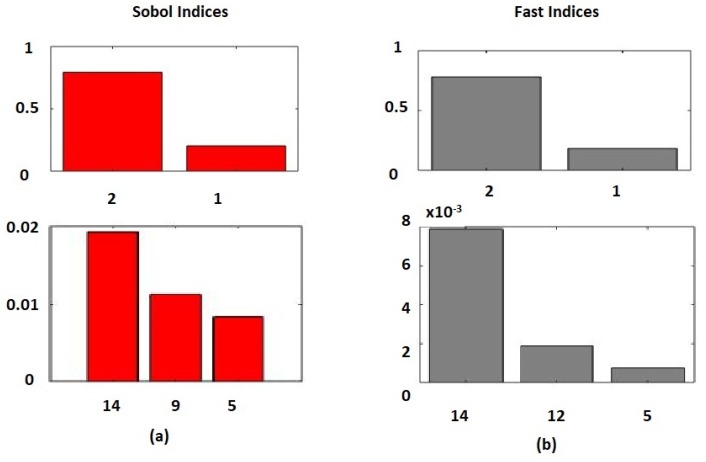
Sobol and FAST sensitivity indices of carbon monoxide.

**Figure 7:**
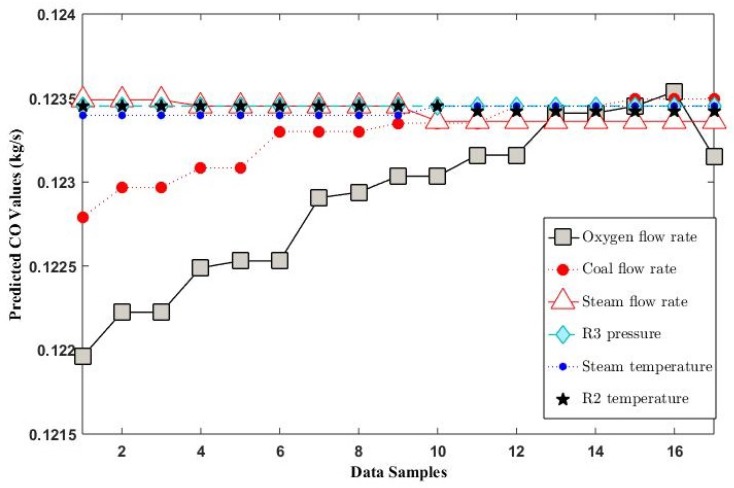
Sensitivity analysis of oxygen flow rate, coal flow rate, steam flow rate, R3 pressure, steam temperature, and R2 temperature through the ensemble model.

Sensitivity analysis was performed using Sobol and FAST methods. In the Sobol method, the top five most sensitive variable were oxygen flow rate, coal flow rate, steam flow rate, the pressure of decomposer unit (R3) and pyrolysis temperature as shown in Figure 6a. In the FAST-based sensitivity analysis, the top five sensitive variables were oxygen flow rate, coal flow rate, steam flow rate, steam temperature, and pyrolysis temperature as shown in Figure 6b. The top three and the fifth ranked variables were common. The only difference in ranking was the variable ranked at number four. In addition to the FAST and Sobol-based sensitivity analysis, the ensemble model was also used to perform sensitivity analysis of the top six sensitive variables indicated in Figure 6. For 5% variation in steady state value of these variables, their effect on mass flow rate of CO is shown in Figure 7.

The first most sensitive variable was O${}_{2}$ flow rate. According to Huang et al., the CO production increases with increase in air flow rate from eight to 12 normal cubic meters per hour. After nine normal cubic meters per hour, the CO formation decreases with increase in oxygen flow rate; the decrease in mass flow rate of CO is due to the formation of CO${}_{2}$ in presence of excessive air [13].

The second most sensitive variable was the flow rate of coal. The production of CO is directly proportional to the flow rate of coal [13]; an increase in the coal flow rate results in a reduction of coal bed temperature, a decrease in CO${}_{2}$ formation, and increase in CO formation.

The third most sensitive variable was steam flow rate. With the increase in the flow rate of steam, the production of CO increases for steam to coal ratio from 0.2 to 0.6. Afterwards, CO mass flow rate decreased due to the conversion of CO to CO${}_{2}$; the CO produced in the gasifier started to react with the steam to produce CO${}_{2}$ [32].

The fourth most sensitive variable in Sobol sensitivity analysis was R3 pressure; it was observed that R3 pressure and formation of CO were directly proportional because of the reaction kinetics where reaction moves in a forward direction with an increase in pressure [33]. The fourth most sensitive variable in the FAST analysis was steam temperature. Increase in steam temperature results in increase in CO formation [13].

The fifth most sensitive variable, in the Sobol as well as FAST based sensitivity analysis, was pyrolysis reactor (R2) temperature. In R2, there is already a presence of CO coming from R1 so with the further increase in temperature from 1050 ${}^{\circ }$C to 1060 ${}^{\circ }$C, the CO converts to CO${}_{2}$ [33].

**Figure 8:**
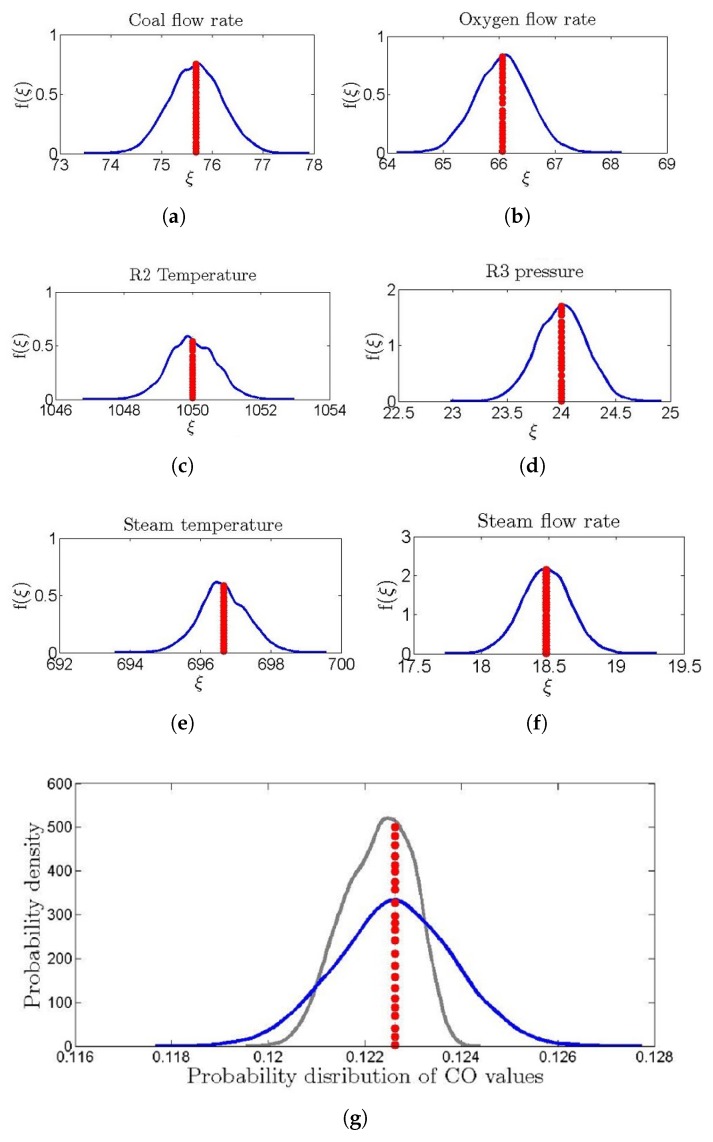
(**a**–**f**) Polynomial chaos expansion (PCE)-based predicted distribution of input variables (blue) and measured value of entrained flow coal gasification (red dotted lines), (**g**) PCE-based derived distribution of measured CO values (blue), non-intrusive PCE-based predicted distribution of CO (grey) and measured value of CO (red dotted line).

The results of non-intrusive PCE-based uncertainty analysis were used to know the effect of uncertainty in input variables on CO mass flow rate. For 0.5% uncertainty in the measured values of all the fourteen input variables, an average of 2% uncertainty was observed in the PCE-based prediction of CO mass flow rate as shown in Figure 8. In order to compare prediction accuracy of the PCE-based method with the ensemble model, mean values of the predictive distributions were determined. Correlation between actual values of CO mass flow rate and mean values of the predictive distributions was 0.90.

**Figure 9:**
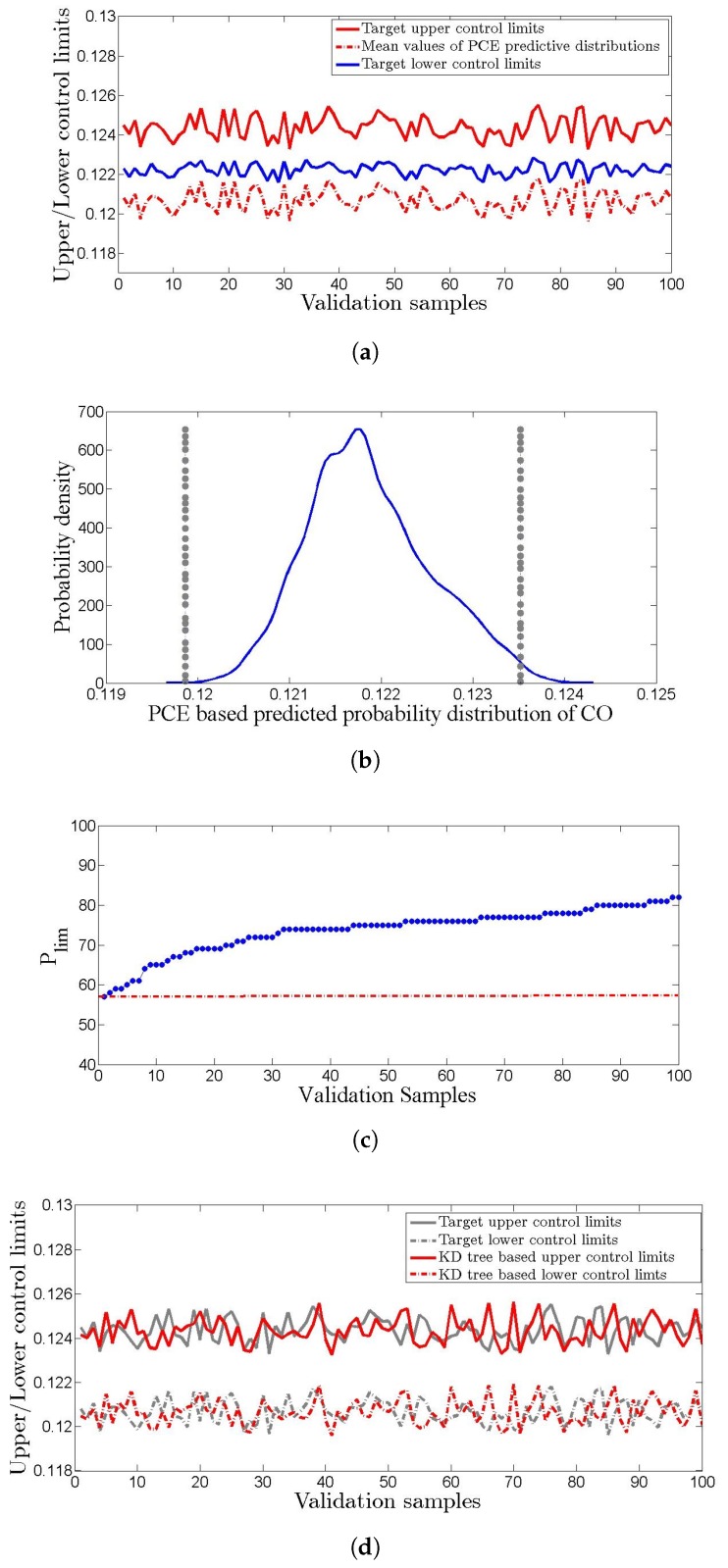
(**a**) Measured data-based control limits and deterministic prediction of the PCE-based method, (**b**) off-line control limits and predictive distribution of CO mass fraction, (**c**) $P_{lim}$ of validation samples, (**d**) measured data-based and *k*-*d* tree-based control limits.

The results of reliability analysis are shown in Figure 9a–d. In the off-line reliability analysis, 4% of the predictive distributions could not qualify the threshold of reliability as shown in Figure 9c. The k–d tree-based derived control limits had 0.90 correlation coefficient with the measured data based control limits, Figure 9d. When the percentage of predictive distribution within the control limits comes below a threshold, a control problem is sensed by the proposed method; it is inferred that uncertainty in one or more input variables is larger than the allowable limit.

## 6. Conclusions

In this work, a novel framework comprised of data-based prediction, sensitivity analysis, uncertainty analysis and reliability analysis of conversion efficiency of entrained flow gasification process was developed. MATLAB^®^–Excel^®^–Aspen^®^ interfacing was used to generate data for development of the modeling framework. Mass flow rate of carbon monoxide in the product stream was assumed as the representative of conversion efficiency. The correlation coefficient between the predicted values of CO mass flow rate and the measured values of CO mass flow rate was 0.95. The top most sensitive variables were oxygen flow rate, coal flow rate, steam flow rate, the pressure of decomposer unit, steam temperature, and pyrolysis temperature. For 0.5% uncertainty in the process variables, an uncertainty of 2% was noted in the conversion efficiency. Data-based control limits were used to evaluate the reliability of predicted probability distribution.

The MATLAB^®^–Excel^®^–Aspen^®^ is used to generate process data because of unavailability of real plant data for this study. However, the ultimate aim of the proposed framework is on-line data-based sensing, sensitivity analysis, uncertainty analysis and reliability analysis of a real time coal gasification process. Hence, in future work, the Aspen based process flowsheet can be replaced by an actual coal gasification plant. 

## Figures and Tables

**Table 1:** Material streams and blocks symbols used in Figure 1.

Symbols	Process Variables	Symbols	Process Variables
S1	Pyrolysis 1 Product	R1	Pyrolysis 1
S2	Pyrolysis 2 Product	R2	Pyrolysis 2
S3	Char stream	R3	Decomposer (SEPELEM)
S4	Gas stream	R4	Combustion unit
S5	Ash stream	R5	Gasifier
S6	Combustion product	M	Mixer
S7	Inlet of gasifier	SEP	Separator
Q1	R1 Energy stream	Q2	R2 Energy stream
Q3	R3 Energy stream		

**Table 2:** List of reactions occuring in pyrolysis, decomposition, combustion, and gasification process.

Chemical Reactions	Rxn No.
**Pyrolysis Process**	
Coal → Char + CO + H_2_ + H_2_O + CO_2_ + CH_4_ + H_2_S + N_2_ + C_6_H_6_	1
**Decomposition process**	
Char → C + H_2_ + O_2_ + N_2_ + S + Ash	2
**Combustion Process**	
C_6_H_6_ + 7.5 O_2_ → 6 CO_2_ + 3 H_2_O	3
H_2_ + 0.5 O_2_ → H_2_O	4
CO + 0.5 O_2_ → CO_2_	5
CH_4_ + 2 O_2_ → CO_2_ + 2 H_2_O	6
**Gasification Process**	
C + O_2_ → CO_2_	7
C + 0.5 O_2_ → CO	8
C + H_2_O → CO + H_2_	9
C + CO_2_ → 2 CO	10
C + 2 H_2_ → CH_4_	11
S + H_2_ → H_2_S	12
H_2_ + 0.5 O_2_ → H_2_O	13
CO + 0.5 O_2_ → CO_2_	14
CH_4_ + 2 O_2_ → CO_2_ + 2 H_2_O	15
CO + H_2_O → CO_2_ + H_2_	16
CH_4_ + H_2_O → CO + 3 H_2_	17

**Table 3:** Process variables used for model development.

No.	Process Variable
1	Coal Flow
2	Oxygen Flow
3	R5 Pressure
4	R4 Pressure
5	R2 Temperature
6	R2 Pressure
7	SEP Pressure
8	R3 Temperature
9	R3 Pressure
10	R1 Temperature
11	R1 Pressure
12	Steam Temperature
13	Steam Pressure
14	Steam Flow

**Table 4:** Process variables used for soft-sensor development.

No.	Process Variables	Units	Values
1	Coal Flow	gm/s	75.68
2	Oxygen Flow	gm/s	66.05
3	R5 Pressure	atm	24
4	R4 Pressure	atm	24
5	R2 Temperature	${}^{\circ }$C	1050
6	R2 Pressure	atm	24
7	SEP Pressure	atm	24
8	R3 Temperature	${}^{\circ }$C	1050
9	R3 Pressure	atm	24
10	R1 Temperature	${}^{\circ }$C	1050
11	R1 Pressure	atm	1
12	Steam Temperature	${}^{\circ }$C	423.51
13	Steam Pressure	atm	24
14	Steam Flow	gm/s	18.48
